# 

**Published:** 1866-06

**Authors:** 


### Books and Pamphlets Received.

Clinical Notes on Uterine Surgery, with special reference to the management of sterile condition. By J. Marion Sims, A. B., M. D. New York: Wm. Wood & Co., 1866.

Asiatic Cholera, its origin and spread in Asia, Africa and Europe; introduction into America through Canada; remote and proximate causes, symptoms and pathology, and the various modes of treatment analyzed. By R. Nelson, M. D. New York: Wm. A. Townsend, 1866.

Godey's Lady's Book for June.

Atlantic Monthly for June.

Cholera—Facts and Conclusions as to its Nature, Prevention and Treatment. By Henry Hartshorne, A. M., M. D. Philadelphia: J. B. Lippincott & Co., 1866.

Reflex Paralysis; its Pathological Anatomy, and relation to the Nervous System. By M. Gonzalez Echeverria, M. D. New York: Baillière Bros., 1866.

Seventeenth Annual Announcement of Female Medical College of Philadelphia, 1866.

The Beacon, or a Warning to Young and Old. By Wm. Cornell, M. D., LL.D. Philadelphia: F. Humphrey & Co., 1865.

Instructions in the Preparation, Administration, and Properties of Nitrous Oxide, Protoxide of Nitrogen, or Laughing Gas. By George D. Barker, D. D. S. Philadelphia: Rubercame & Stockton, 1866.

Cholera: its Characteristics, History, Treatment, Geographical Distribution of Different Epidemics, Suitable Sanitary Preventions, etc. Illustrated with Lithographic Map and Microscopic Drawings. By William B. Fletcher, M. D. Cincinnati: Robert Clarke & Co., 1866.

The Lower Depths of the Great American Metropolis. A Discourse by the Rev. Peter Stryker. New York: Schermerhorn, Bancroft & Co., 1866.

American Literary Messenger for June.

Erie County Medical Society.—The regular Annual Meeting of the Society is held on the second Tuesday of January, at half past ten o'clock A. M., at the rooms of the Buffalo Medical Association, Young Men's Association Buildings; and the Semi-Annual Meeting is held on the second Tuesday of June, at the same time and place.

